# Development and internal validation of a machine learning–based prediction model for pulmonary hypertension in COPD

**DOI:** 10.3389/fmed.2026.1752113

**Published:** 2026-02-18

**Authors:** Ruoyu Wang, Jie Tan, Guangping Li, Zhenyu Pan, Huiling Guo, Wei Sun, Jing Wang

**Affiliations:** 1 Department of Respiratory and Critical Care Medicine, Beijing Chaoyang Hospital, Capital Medical University, Beijing, China; 2 School of Information Engineering, Guangdong University of Technology, Guangzhou, China; 3 Department of Radiology, Beijing Chaoyang Hospital, Capital Medical University, Beijing, China

**Keywords:** CatBoost algorithm, chronic obstructive pulmonary disease, clinical prediction model, feature selection, machine learning, pulmonary hypertension, SHAP

## Abstract

**Background:**

Chronic obstructive pulmonary disease (COPD) is frequently complicated by pulmonary hypertension (PH), which worsens prognosis, but early PH detection is limited by the invasiveness or suboptimal sensitivity of current diagnostic tools.

**Methods:**

In this retrospective study, we analyzed 523 hospitalized patients with COPD from Beijing Chaoyang Hospital. After standardized preprocessing and recursive feature elimination, 18 routinely available noninvasive clinical and physiological variables were retained as predictors. Eight machine-learning algorithms were trained to predict PH and compared using area under the receiver operating characteristic curve (AUC), accuracy, sensitivity, specificity, F1 score, and decision-curve analysis; model interpretability was assessed with Shapley additive explanations (SHAP).

**Results:**

The CatBoost model showed the best discrimination (AUC 0.848; accuracy 0.830; sensitivity 0.758; specificity 0.866; F1 0.746). SHAP analysis identified right ventricular diameter, pulmonary artery diameter, arterial partial pressure of carbon dioxide, right atrial transverse diameter, and age as the most influential predictors.

**Conclusion:**

A CatBoost-based prediction model using readily obtainable noninvasive variables can estimate PH risk in COPD with good accuracy and provide transparent feature-level explanations, potentially facilitating earlier detection and risk-stratified management.

## 1 Introduction

### 1.1 Disease burden in COPD

Chronic obstructive pulmonary disease (COPD) causes long-term blockage of airflow and is a major cause of illness and death, and it greatly reduces quality of life. Long-lasting inflammation in the airways and changes in airway structure are common, and pulmonary hypertension (PH) is another important factor that affects outcome (1, 2). The link between COPD and PH comes from several processes. These include changes in the lung blood vessels, inflammatory responses driven by cytokine signals, and the control of genetic risk. Lung tissue (parenchymal) damage relates to how often PH occurs and how severe it is, but there is a lot of variation between patients: some people with only mild changes in the lungs develop severe PH, while others with advanced disease have only small increases in pulmonary artery pressure (2–4). This mismatch between lung tissue damage and blood vessel changes suggests that changes inside the vessels, inflammatory mediators, and genetic factors may all work together to cause PH in COPD, even when parenchymal disease is not very severe (5, 6).

### 1.2 Clinical importance of PH in COPD

Because of these differences between patients and their effects on outcome, it is very important to tell apart pulmonary hypertension and non-pulmonary hypertension in people with chronic obstructive pulmonary disease. This is important because it affects treatment plans, decisions about who needs urgent care, and how often patients should be checked (7). Early detection enables timely, targeted interventions, which are associated with a reduction in hospitalizations and an improvement in quality of life (8). Precise differentiation between COPD with and without PH also supports phenotype-informed treatment strategies (9). Despite its clinical importance, PH is still frequently missed or recognized late in patients with COPD.

### 1.3 Diagnostic limitations in detecting PH

Early detection of PH in COPD is hampered by nonspecific symptoms such as exertional dyspnea and chest tightness, which are difficult to attribute to vascular versus airway pathology and often delay further investigation. Delayed diagnosis is associated with worse outcomes (10, 11). Right heart catheterization remains the diagnostic gold standard, but its invasiveness and resource requirements limit use in routine practice (12–14). Echocardiography provides noninvasive assessment of right-heart structure and pressure surrogates but cannot deliver continuous monitoring and may be constrained by hyperinflation-related acoustic windows (15, 16). In low- and middle-income countries, shortages of trained personnel and equipment further contribute to underdiagnosis and fragmented longitudinal management (17). Several inexpensive and widely available biomarkers—such as mean platelet volume (MPV), red blood cell distribution width (RDW), brain natriuretic peptide (BNP), the pulmonary artery–to–aortic diameter ratio, and the neutrophil-to-lymphocyte ratio (NLR)—have been associated with PH risk in COPD (18–21). But when these biomarkers are used alone, they do not have enough power to clearly separate patients into different risk groups. Because of this problem, researchers are studying machine learning (ML) methods that bring together different kinds of data and improve diagnostic accuracy.

### 1.4 Advances in machine learning for COPD–PH detection

ML methods can use many types of data in COPD at the same time, such as clinical features, pulmonary function tests, vascular indicators from CT, and blood biomarkers. By using these different inputs together, ML models can find complex risk patterns. With these high-dimensional data, ML models can diagnose disease more accurately and more completely than methods that rely on fixed rules or a single measurement, and they can give quantitative support for decisions in clinical practice (22, 23). Recent studies show that ML can increase the detection rate of pulmonary hypertension in patients with chronic lung disease, but reliable and easy-to-explain tools for pulmonary hypertension related to COPD are still not available.

### 1.5 Research gaps and study objectives

ML-based methods may help provide individualized risk assessment and improve the accuracy of detecting PH in patients with COPD. But there are still questions about whether these methods work well in many different clinical settings, how clear their decisions are, and how easily they can be added to daily clinical work (24, 25). Solving these problems is important for safe and wide use in clinical practice. Accordingly, this study aimed to develop and internally validate an interpretable ML-based risk-prediction model for PH in COPD. The model is designed to (a) estimate individualized PH risk, (b) clarify feature-level relationships between COPD and PH, and (c) support phenotype-informed therapeutic planning. The intended end-users are respiratory and cardiovascular specialists, and the intended use is early in-hospital risk screening and referral stratification among hospitalized patients with COPD, to help determine whether further advanced examinations, such as echocardiography or right heart catheterization are warranted.

## 2 Methods

### 2.1 Study design and setting

This single-center, retrospective, cross-sectional observational study was conducted at Beijing Chaoyang Hospital. We analyzed inpatients with COPD, with and without PH, admitted to the Department of Respiratory and Critical Care Medicine between January 2014 and September 2024. Clinical and laboratory data were obtained from the hospital electronic medical record system. The healthcare setting, eligibility criteria, outcome definition, and predictor measurements were identical for participants later allocated to the training and test sets.

### 2.2 Study participants

Eligible participants were adults (aged 18–95 years) with a diagnosis of COPD confirmed by pulmonary function testing in accordance with Global Initiative for Chronic Obstructive Lung Disease (GOLD) guidelines.

We excluded patients with: (a) idiopathic pulmonary arterial hypertension (PAH), pulmonary thromboembolism (PTE), or PH associated with congenital heart disease; (b) severe cardiovascular or cerebrovascular disease, or hepatic or renal insufficiency; or (c) active malignancy. For patients with multiple hospitalizations during the study period, only the first eligible admission was considered to avoid duplicate observations.

All consecutive patients meeting the criteria during the accrual period were included, yielding a total of 523 hospitalized patients with COPD, of whom 176 (33.6%) met diagnostic criteria for COPD-PH (i.e., outcome events). According to commonly cited standards for multivariable prediction models, an events-per-predictor (EPV) ratio of at least 10 is often recommended to reduce overfitting and enhance parameter stability. In the present study, the final CatBoost model included 18 predictors and 176 outcome events, corresponding to an EPV of approximately 9.8, which is close to this conventional target and exceeds the minimum EPV threshold of 5 that some methodological work and guidelines still consider acceptable. This EPV suggests that the available data were adequate to support model development and internal validation.

### 2.3 Definitions

#### 2.3.1 COPD definition

In accordance with GOLD recommendations, COPD was defined as a post-bronchodilator ratio of forced expiratory volume in 1 s to forced vital capacity (FEV₁/FVC) < 0.70 on spirometry, with FEV₁ and FVC measured using standard post-bronchodilator protocols (26).

#### 2.3.2 PH definition

(1) Right heart catheterization (RHC): PH was diagnosed when resting mean pulmonary arterial pressure (mPAP) > 20 mm Hg, in line with contemporary ESC/ERS guidance; 43 patients met this definition. All 43 also fulfilled the echocardiographic criteria below (27).

(2) Transthoracic echocardiography: In the absence of RHC, PH was considered present when transthoracic echocardiography showed a peak tricuspid regurgitant velocity (TRV) > 2.8 m/s; an additional 133 patients met this criterion (excluding the 43 RHC-diagnosed cases to avoid double counting).

Patients who did not meet either criterion (1) or (2) were classified as non-PH. Transthoracic echocardiographic measurements were performed and reported by board-certified sonographers, and RHC was undertaken by interventional cardiologists according to contemporary guidelines. As all investigations were performed as part of routine clinical care in this retrospective cohort, assessors were not formally blinded to other clinical information or to the study hypothesis.

### 2.4 Predictors and data handling

Guided by expert consensus and prior evidence, 39 candidate predictors were prespecified and grouped into six domains: demographics, personal history, medical history, laboratory indices, lung function, and echocardiographic parameters (Table 1). TRV was excluded *a priori* from candidate predictors because it formed part of the echocardiographic outcome definition (TRV > 2.8 m/s), thereby avoiding incorporation bias.

**Table 1:** Characteristic variables.

Category	Variables
Demographics	Gender, age, height, weight, body mass index (BMI)
Personal history	Smoking index (average number of cigarettes smoked per day × total years of smoking), smoking cessation years
Medical history	Diabetes mellitus, hyperlipidemia
Laboratory	Blood gases: oxygen partial pressure (PaO_2_), carbon dioxide partial pressure (PaCO_2_)
	Biochemical tests: creatinine (Cr), uric acid (UA), albumin (ALB)
	Inflammatory markers: C-reactive protein (CRP), erythrocyte sedimentation rate (ESR)
	Routine blood tests: mean platelet volume (MPV), red blood cell distribution width-coefficient of variation (RDW-CV), platelet count (PLT), white blood cell count (WBC), hemoglobin content (HB), neutrophil count (NE), lymphocyte count (LY), eosinophil count (EO), neutrophil/lymphocyte ratio (NLR)
	Cardiac function indicators: N-terminal B-type natriuretic peptide precursor (NT-proBNP)
Lung function	Forced expiratory volume in one second/forced vital capacity (FEV_1_/FVC), predicted forced expiratory volume in one second (FEV_1_% predicted, FEV_1_pred%), predicted forced vital capacity (FVC% predicted, FVCpred%), diffusion capacity of carbon monoxide (DLCO SB)
Echocardiography	Chamber sizes: right atrial transverse diameter (RA TD), right atrial longitudinal diameter (RA LD), right ventricular diameter (RVD), pulmonary artery diameter (PA), ascending aortic diameter (AAo)
	Function: ejection fraction (EF), interventricular septal thickness (IVS)
	Hemodynamics: pulmonary valve peak systolic flow velocity (PVmax), and pulmonary artery diameter to ascending aortic diameter ratio (PA/AO)

After random shuffling, the 523 patients were split into a training set (*n* = 423) and an independent test set (*n* = 100) in an 8:2 ratio. Model selection and stability were assessed using stratified five-fold cross-validation within the training set. The optimal model was then refitted on the full training set and evaluated on the test set. Decision thresholds for all performance metrics were fixed during cross-validation and were not adjusted on the test set.

**Figure 1:**
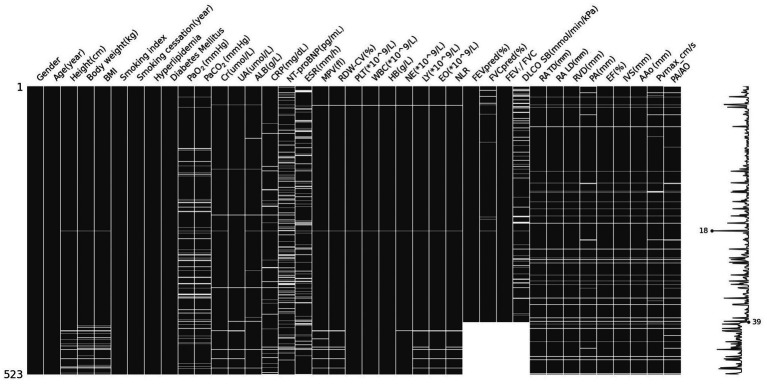
Missing data pattern for the 39 candidate predictors in 523 patients.

Variables with less than 30% missing values were filled in using the k-nearest neighbors (kNN) method. Missing values for each variable are shown in Supplementary Table 1 and in Figure 1. In Figure 1, each column shows one variable, and white cells show missing values. Variables with 30% or more missing values were not used in model development.

To deal with class imbalance, we used synthetic oversampling of the minority class (SMOTE) on the training data in each cross-validation fold before model fitting. This method lowers bias toward the majority class and keeps the original feature space.

### 2.5 Feature selection

**Figure 2:**
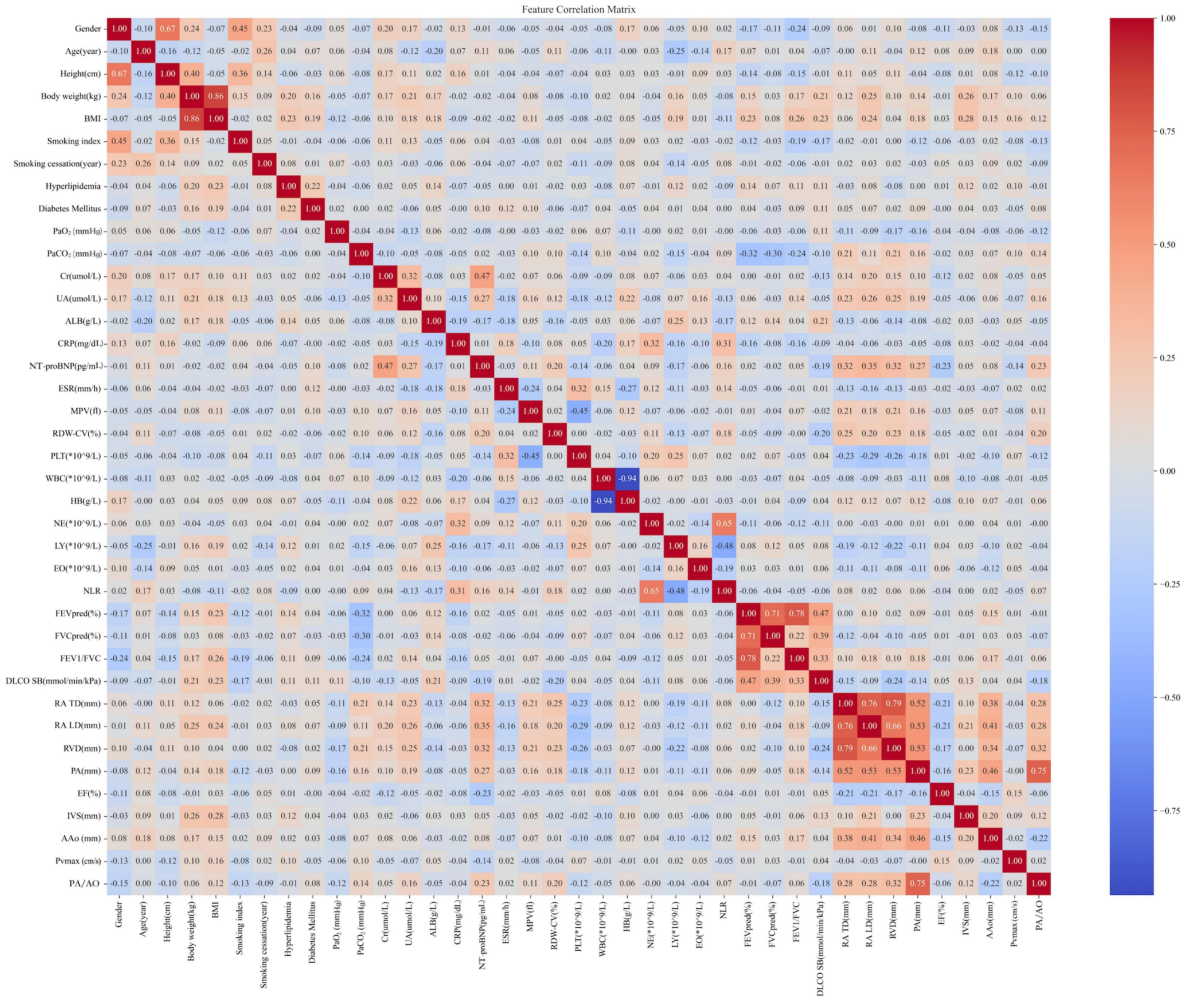
Correlation heatmap for the 39 candidate predictors.

The initial 39 candidate predictors underwent multicollinearity testing, visualized using a correlation matrix to generate a heatmap (Figure 2). Features with an absolute pairwise Pearson correlation coefficient (|r|) > 0.80 were removed to limit redundancy and reduce estimator instability. The |r| = 0.80 threshold was chosen as a pragmatic, commonly used cutoff in applied epidemiology and machine-learning studies to flag near-duplicate variables while preserving clinically distinct information, thereby balancing model stability and information retention (28).

Recursive feature elimination (RFE) was then applied to the pruned feature set. All correlation-based screening and RFE procedures were performed within each training fold and applied only to the corresponding held-out fold to avoid information leakage. Based on these procedures, a final set of 18 predictors was selected and subsequently used for model training in the full training set and performance evaluation in the independent test set.

### 2.6 Model development

Before model fitting, continuous predictors were standardized using z-score transformation, and binary or categorical variables were encoded as dummy (one-hot) indicators for all algorithms except CatBoost, which used its native handling of categorical features. A unified machine-learning pipeline was applied to the retained predictors using eight algorithms: Categorical Boosting (CatBoost), Random Forest (RF), Gradient Boosting Machine (GBM), Adaptive Boosting (AdaBoost), Logistic Regression (LR), Extreme Gradient Boosting (XGBoost), k-Nearest Neighbors (kNN), and Multilayer Perceptron (MLP).

Within this pipeline, data preprocessing and grid-search hyperparameter optimization were carried out prior to final model fitting; algorithm-specific hyperparameters are summarized in Supplementary Table 2.

### 2.7 Model evaluation

Model performance was evaluated using stratified five-fold cross-validation in the training set and subsequently in the independent test set. Discrimination was summarized using the receiver operating characteristic (ROC) curve and area under the curve (AUC). For threshold-based performance, we reported accuracy, sensitivity, specificity, F1 score, positive predictive value (PPV), and negative predictive value (NPV).

For each model, performance across the five validation folds was summarized as the mean and 95% confidence interval (95% CI), providing estimates of central tendency and sampling variability. Each model produced an individual-level predicted probability of COPD-PH.

We used decision curve analysis (DCA) to measure the possible clinical benefits over a range of reasonable intervention thresholds. In addition, we measured the marginal contribution of each predictor variable to the model output by calculating SHAP values (Shapley Additive Explanations). We did this at the cohort level and at the individual patient level. This helped make the model easier to understand in clinical practice.

### 2.8 Statistical analysis

All statistical analyses were done in R (version 4.4.1) and Python (version 3.12.4). We used R mainly for data management and standard statistical analyses. We used the packages readxl (for data import), dplyr (for data handling), boot (for resampling-based inference), and effsize (for effect size estimation). We used Python to compute SHAP values and plots to explain features in the prediction models.

We compared basic features between the training set and the test set with the Mann–Whitney U test. For categorical variables we used Fisher’s exact test. We set the significance level at *α* = 0.05. By checking similarity in key covariates between the training and test sets, we assessed possible selection bias and supported the model performance estimates.

We then used Cohen’s d for paired samples to measure the practical size of differences in predictive performance between models. We estimated its 95% confidence interval with 1,000 non-parametric resampling runs. We interpreted the effect size with common cut points: |d| < 0.2 (no practical difference), 0.2 ≤ |d| < 0.5 (small), 0.5 ≤ |d| < 0.8 (moderate), and |d| ≥ 0.8 (large). A larger absolute value means a stronger practical effect (29).

## 3 Results

### 3.1 Patient characteristics

After application of the inclusion and exclusion criteria, 523 hospitalized patients with COPD were included in the analysis. Supplementary Table 3 presents baseline characteristics for the training and test sets, and Table 2 compares patients with COPD-PH and those with COPD alone.

**Table 2:** Baseline characteristics of patients with COPD-PH and COPD alone.

Characteristic	COPD	COPD-PH	*p* value
	*N* = 347	*N* = 176	
Female (*n*)	94 (27%)	59 (34%)	0.2
Male (*n*)	253 (73%)	117 (66%)	0.2
Age (years)	68 ± 9	70 ± 10	0.012
Height (cm)	165.5 (160.0, 171.0)	164.0 (159.5, 170.0)	0.079
Body weight (kg)	64.0 (57.1, 72.0)	63.0 (55.0, 71.0)	0.041
BMI	23.5 (21.4, 26.1)	23.1 (19.5, 26.3)	0.2
Smoking index	5 (0, 8)	3 (0, 8)	0.03
Smoking cessation (years)	0 (0, 5)	0 (0, 5.3)	0.7
History of hyperlipidemia (*n*)	46 (13%)	13 (7%)	0.063
No history of hyperlipidemia (*n*)	301 (87%)	163 (93%)	0.063
History of diabetes mellitus (*n*)	69 (20%)	33 (19%)	0.8
No history of diabetes mellitus (*n*)	278 (80%)	143 (81%)	0.8
PaO_2_ (mm Hg)	75.3 (65.7, 84.3)	66.2 (55.9, 85.5)	0.2
PaCO_2_ (mm Hg)	41.0 (38.0, 44.7)	46.9 (38.9, 57.3)	<0.001
Cr (μmol/L)	69.0 (60.2, 80.6)	70.5 (59.4, 83.5)	0.4
UA (μmol/L)	307.9 (245.1, 386.4)	327.1 (246.2, 420.0)	0.003
ALB (g/L)	38.0 (35.8, 40.2)	37.0 (34.4, 39.3)	0.025
CRP (mg/dL)	5.0 (0.9, 7.9)	1.7 (0.5, 5.0)	0.016
NT-proBNP (pg/mL)	111.0 (44.3, 263.6)	371.0 (95.0, 2205.0)	<0.001
ESR (mm/h)	15.0 (5.0, 34.8)	10.0 (5.0, 25.0)	0.021
MPV (fL)	9.9 (9.2, 10.6)	10.4 (9.6, 11.1)	<0.001
RDW-CV (%)	13.3 (12.8, 13.9)	13.8 (12.9, 14.6)	<0.001
PLT ( × 10^9^/L)	213.0 (173.5, 260.5)	190.0 (154.0, 233.0)	<0.001
WBC ( × 10^9^/L)	7.6 (5.7, 10.5)	6.9 (5.3, 10.5)	0.089
HB (g/L)	130.0 (102.0, 142.0)	129.5 (95.5, 146.0)	0.072
NE ( × 10^9^/L)	3.9 (3.0, 5.4)	4.2 (3.1, 5.6)	>0.9
LY ( × 10^9^/L)	1.6 (1.3, 2.1)	1.3 (0.9, 1.9)	<0.001
EO ( × 10^9^/L)	0.2 (0.1, 0.2)	0.1 (0.1, 0.2)	0.006
NE/LY	2.4 (1.7, 3.6)	3.0 (2.1, 5.0)	0.008
FEV_1_pred (%)	59.0 (39.4, 76.0)	47.5 (30.3, 68.9)	0.001
FVCpred (%)	90.1 ± 20.1	76.3 ± 23.0	<0.001
FEV_1_/FVC	51.0 (37.0, 61.0)	51.2 (38.0, 61.0)	>0.9
DLCO SB (mmol/min/kPa)	71.6 ± 21.6	50.9 ± 22.4	<0.001
RA TD (mm)	32.0 (29.0, 35.0)	37.5 (32.0, 44.0)	<0.001
RA LD (mm)	43.0 (41.0, 47.0)	49.5 (44.3, 54.0)	<0.001
RVD (mm)	30.0 (28.0, 33.0)	36.0 (31.0, 42.0)	<0.001
PA (mm)	23.0 (21.0, 25.0)	27.5 (23.0, 30.9)	<0.001
EF (%)	68.0 (65.0, 71.0)	67.5 (63.0, 70.0)	<0.001
IVS (mm)	10.0 (9.2, 10.6)	10.0 (9.0, 10.7)	>0.9
AAo (mm)	31.0 (29.0, 34.0)	33.0 (30.0, 36.0)	<0.001
PVmax (cm/s)	88.5 (79.8, 100.3)	91.5 (76.0, 105.3)	0.6
PA/AO	0.7 (0.7, 0.8)	0.8 (0.7, 0.9)	<0.001
TRV (m/s)	2.20 (2.01, 2.50)	3.75 (3.28, 4.28)	<0.001

**Figure 3:**
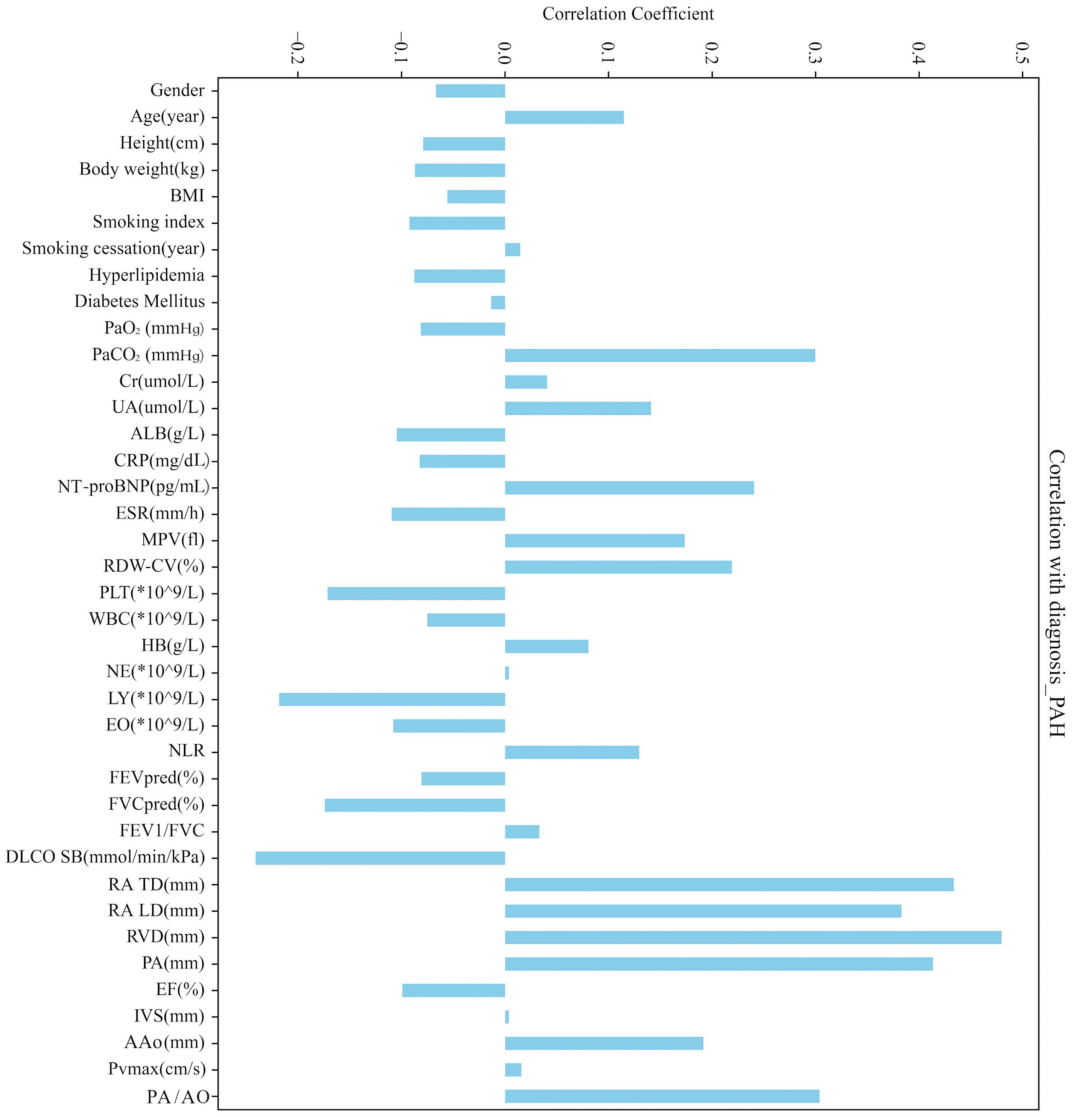
Correlations between the 39 candidate predictors and COPD-PH status.

Correlation analysis between the 39 candidate predictors and COPD-PH status (Figure 3) highlighted right-heart dimensional indices and central pulmonary arterial measures as the most informative features—specifically right ventricular end-diastolic diameter (RVD), right atrial transverse diameter (RA TD), pulmonary artery diameter (PA), right atrial longitudinal diameter (RA LD), and the pulmonary artery-to-aorta diameter ratio (PA/AO).

Across 40 baseline variables, 36 (90.0%) showed no statistically significant difference between the training and test sets (*p* ≥ 0.05; Supplementary Table 3). The four variables with statistically significant differences were smoking index, NT-proBNP, RA TD, and RVD. For RA TD and RVD, the median differences were both 1 mm (approximately 3% relative difference), with interquartile range (IQR) overlap coefficients of 0.67 and 0.75, respectively, indicating highly overlapping distributions and minimal differences in magnitude. NT-proBNP showed an approximately 12% relative difference in medians and smoking index about 40%; nonetheless, IQR overlap remained substantial (0.52 and 0.80, respectively).

Of the 523 included patients, 176 (33.6%) had COPD-PH and 347 (66.4%) had COPD alone. After random allocation, the training set comprised 423 patients (143 COPD-PH, 280 COPD alone), and the independent test set comprised 100 patients (33 COPD-PH, 67 COPD alone). Overall, the distributions of key predictors and outcomes were similar between the training and test sets, with only small differences in a few variables (e.g., smoking index, NT-proBNP, RA TD, and RVD), supporting the comparability of the development and evaluation datasets.

Among the 176 COPD-PH patients, 133 (75.6%) were diagnosed by echocardiography and 43 (24.4%) were confirmed by RHC. Baseline characteristics were partly different between the RHC-diagnosed and echo-diagnosed subgroups (Supplementary Table 4). In general, the RHC-diagnosed subgroup showed features consistent with greater disease severity and right-heart/pulmonary-artery remodeling (e.g., lower PaO₂ and DLCO and higher NT-proBNP and uric acid, together with larger right-heart or pulmonary-artery dimensions), which likely reflects catheterization referral patterns rather than contradictory diagnostic definitions.

**Figure 4:**
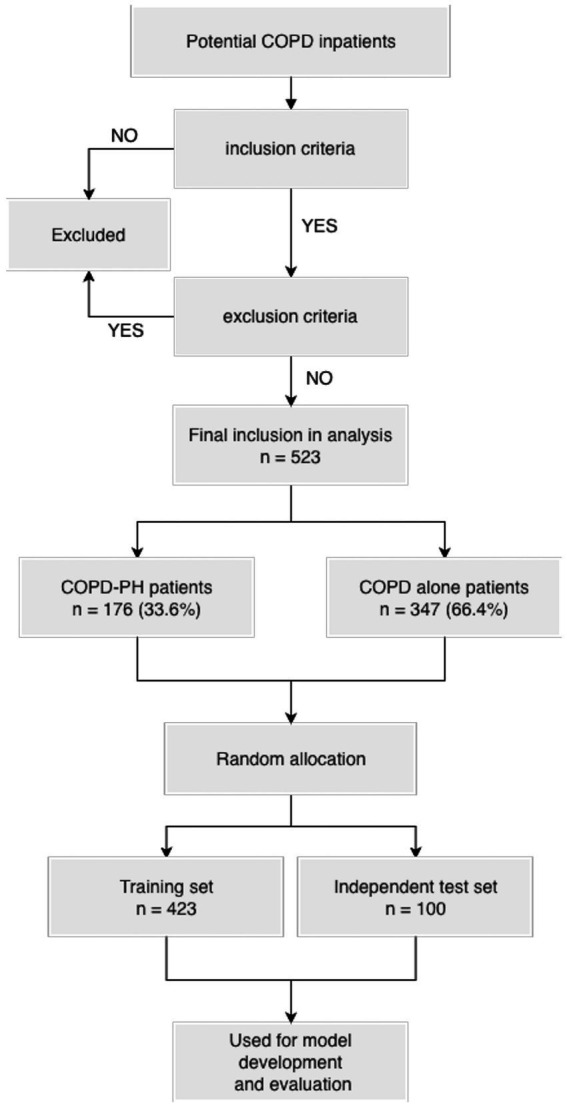
Flow diagram of patient screening, exclusions, and inclusion in the analysis dataset.

The flow of participants through the study, including numbers screened, excluded, and finally included with and without COPD-PH, is shown in Figure 4.

### 3.2 Model development and performance comparison

**Figure 5:**
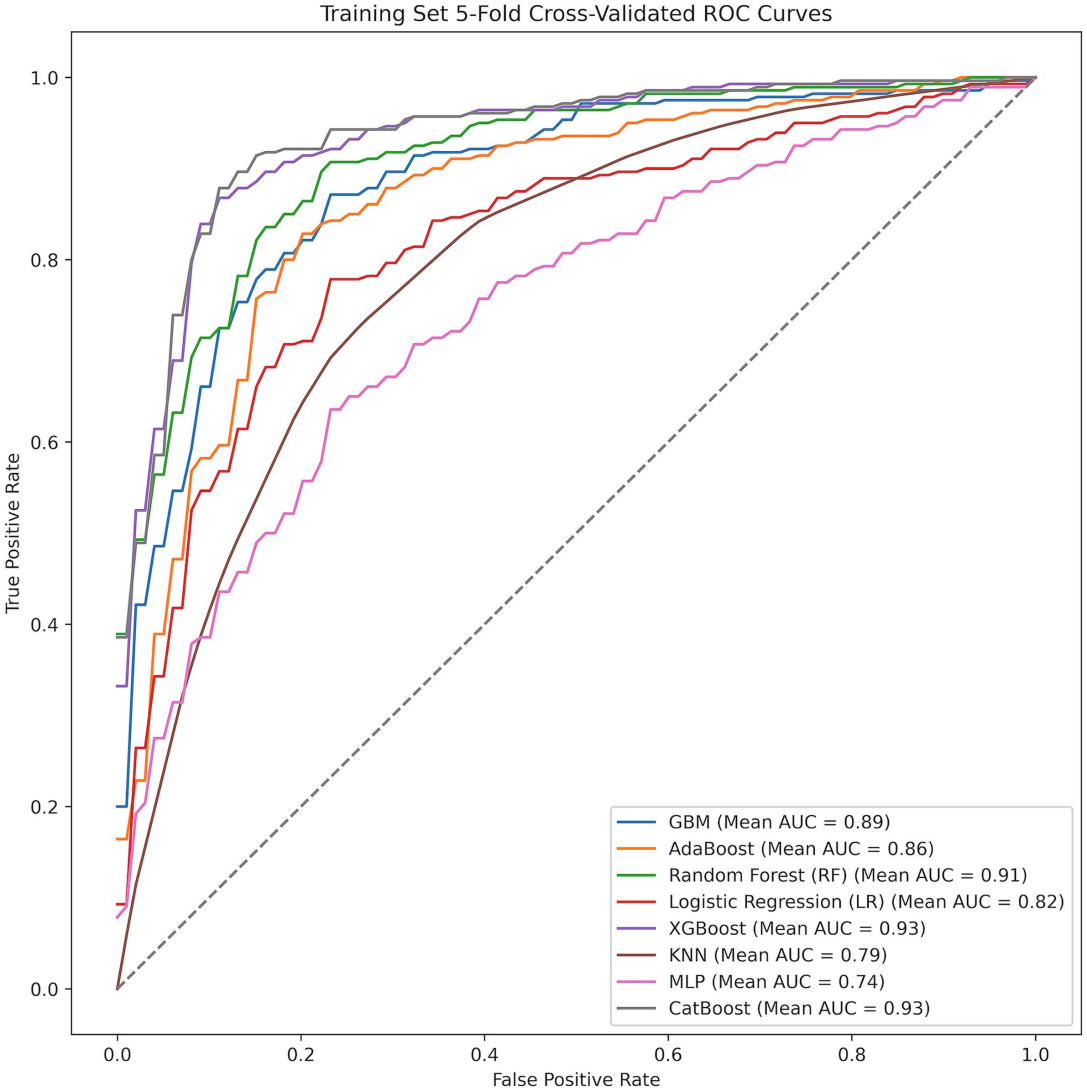
ROC curves from five-fold cross-validation in the training set.

Eight candidate models were compared using seven core performance metrics. Overall cross-validated performance in the training set is summarized in Table 3, and fold-specific results are provided in Supplementary Table 5. The ROC curves from five-fold cross-validation in the training set are shown in Figure 5, illustrating the consistency of discriminative performance across validation folds.

**Table 3:** Overall performance of the candidate algorithms in the training folds.

Model	AUC	Accuracy	Sensitivity (Recall)	Specificity	PPV (Precision)	NPV	F1 Score
MLP	0.745 (95% CI: 0.681, 0.797)	0.662 (95% CI: 0.634, 0.709)	0.739 (95% CI: 0.514, 0.907)	0.586 (95% CI: 0.379, 0.814)	0.689 (95% CI: 0.593, 0.793)	0.742 (95% CI: 0.653, 0.815)	0.669 (95% CI: 0.567, 0.724)
kNN	0.794 (95% CI: 0.778, 0.809)	0.734 (95% CI: 0.714, 0.755)	0.782 (95% CI: 0.757, 0.807)	0.686 (95% CI: 0.625, 0.743)	0.716 (95% CI: 0.684, 0.748)	0.759 (95% CI: 0.747, 0.772)	0.747 (95% CI: 0.737, 0.763)
Logistic Regression (LR)	0.819 (95% CI: 0.788, 0.853)	0.757 (95% CI: 0.707, 0.805)	0.739 (95% CI: 0.671, 0.814)	0.775 (95% CI: 0.718, 0.821)	0.767 (95% CI: 0.716, 0.810)	0.752 (95% CI: 0.694, 0.810)	0.751 (95% CI: 0.698, 0.805)
AdaBoost	0.865 (95% CI: 0.833, 0.891)	0.805 (95% CI: 0.786, 0.830)	0.821 (95% CI: 0.793, 0.850)	0.789 (95% CI: 0.743, 0.846)	0.799 (95% CI: 0.764, 0.840)	0.816 (95% CI: 0.795, 0.837)	0.809 (95% CI: 0.793, 0.832)
GBM	0.885 (95% CI: 0.868, 0.901)	0.814 (95% CI: 0.798, 0.829)	0.839 (95% CI: 0.807, 0.871)	0.789 (95% CI: 0.754, 0.818)	0.800 (95% CI: 0.779, 0.821)	0.833 (95% CI: 0.806, 0.859)	0.819 (95% CI: 0.804, 0.834)
Random Forest (RF)	0.909 (95% CI: 0.887, 0.928)	0.836 (95% CI: 0.818, 0.854)	0.836 (95% CI: 0.800, 0.871)	0.836 (95% CI: 0.811, 0.861)	0.837 (95% CI: 0.814, 0.855)	0.837 (95% CI: 0.809, 0.868)	0.835 (95% CI: 0.815, 0.855)
XGBoost	0.931 (95% CI: 0.904, 0.952)	0.868 (95% CI: 0.838, 0.902)	0.900 (95% CI: 0.875, 0.925)	0.836 (95% CI: 0.789, 0.879)	0.847 (95% CI: 0.811, 0.883)	0.893 (95% CI: 0.867, 0.921)	0.872 (95% CI: 0.844, 0.904)
CatBoost	0.934 (95% CI: 0.908, 0.955)	0.870 (95% CI: 0.845, 0.893)	0.893 (95% CI: 0.875, 0.907)	0.846 (95% CI: 0.796, 0.893)	0.856 (95% CI: 0.816, 0.894)	0.888 (95% CI: 0.873, 0.903)	0.873 (95% CI: 0.851, 0.894)

**Figure 6:**
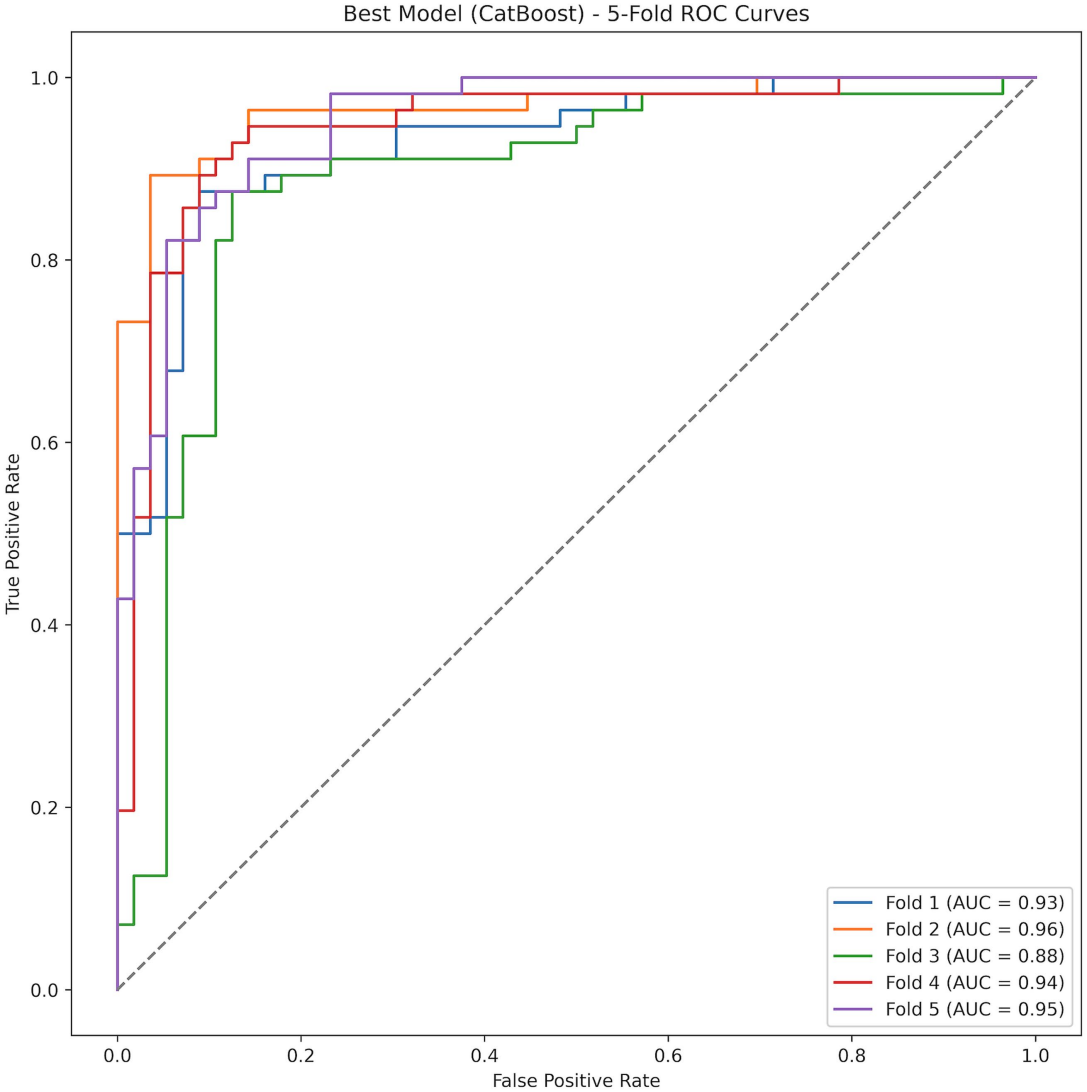
Fold-specific ROC curves for the CatBoost model.

CatBoost demonstrated the best overall internal performance among the eight algorithms. Its fold-specific ROC curves are shown in Figure 6 and indicate stable discrimination across validation folds. In cross-validation, CatBoost achieved an average AUC of 0.9337, accuracy of 0.8696, and F1 score of 0.8732.

Across 196 pairwise model comparisons, effect size analysis using Cohen’s d showed large effects (|d| ≥ 0.8) versus traditional baselines (kNN, MLP), corresponding to 15–31% relative improvements in AUC and accuracy. In contrast, effect sizes versus XGBoost were mostly small or negligible (|d| < 0.5), suggesting broadly comparable predictive performance, although CatBoost was more efficient and easier to tune in practice. Among 49 valid pairwise comparisons favoring CatBoost, 81.6% showed medium-to-large and 75.5% large effects, supporting its selection as the primary model for subsequent test-set evaluation.

**Figure 7:**
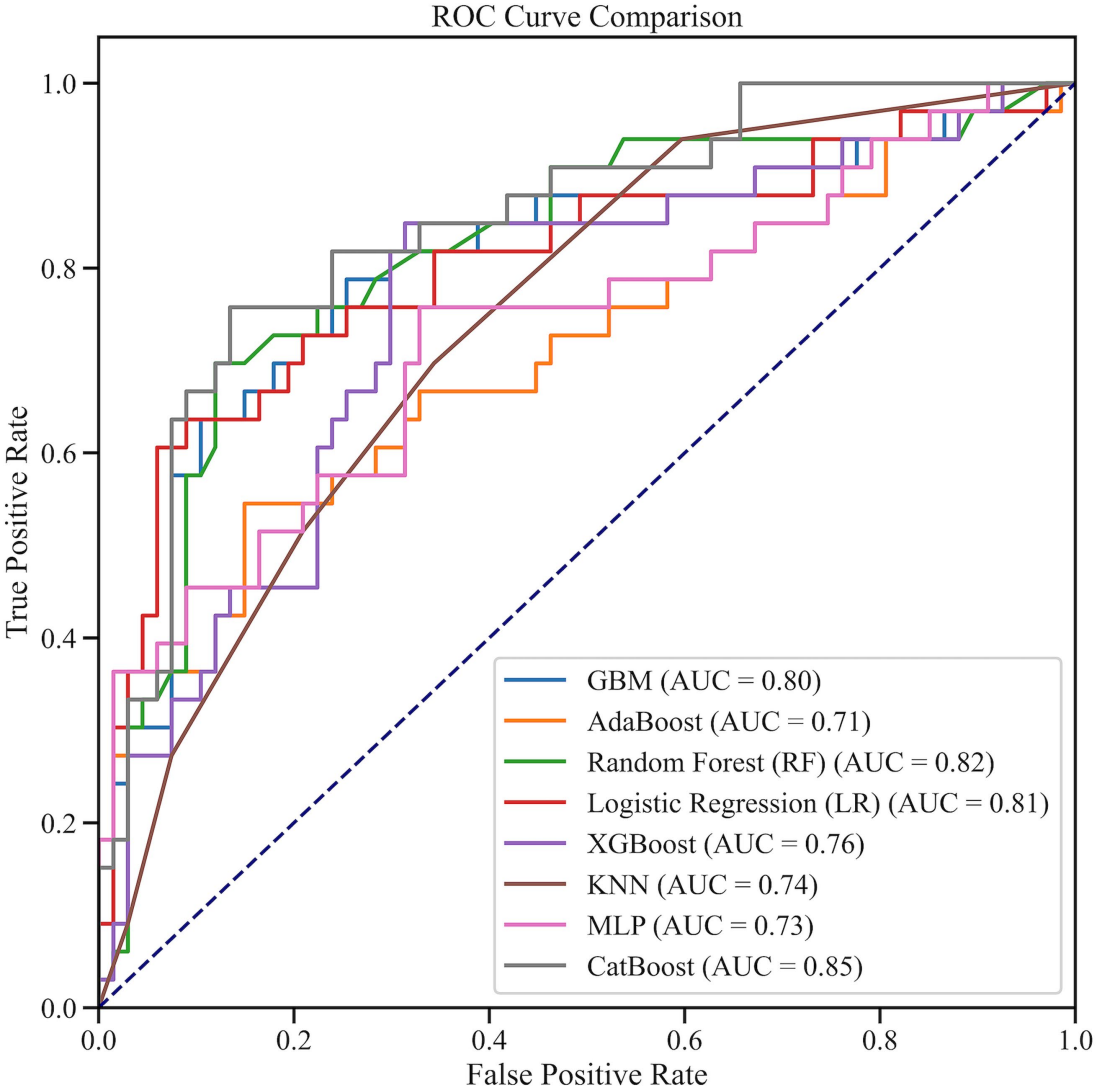
Receiver operating characteristic (ROC) curves for all candidate models on the test set.

On the independent test set, using the decision threshold fixed during cross-validation, CatBoost maintained superior out-of-sample performance (Figure 7). Test-set results for all eight models are summarized in Table 4. CatBoost achieved an AUC of 0.848, accuracy of 0.830, F1 score of 0.746, sensitivity of 0.758, and specificity of 0.866, with balanced PPV and NPV. Random Forest ranked second (AUC 0.815, accuracy 0.820, F1 0.719), while logistic regression (AUC 0.806, F1 0.700) and GBM (AUC 0.804, F1 0.684) showed moderate performance; XGBoost was comparatively weaker (AUC 0.756, F1 0.683).

**Table 4:** Test-set performance of the eight machine-learning models for predicting PH in COPD.

Model	AUC	Accuracy	Sensitivity (Recall)	Specificity	PPV (Precision)	NPV	F1 Score	Best Threshold
GBM	0.804 (95% CI: 0.707, 0.898)	0.760 (95% CI: 0.680, 0.840)	0.788 (95% CI: 0.647, 0.917)	0.746 (95% CI: 0.643, 0.841)	0.605 (95% CI: 0.458, 0.750)	0.877 (95% CI: 0.784, 0.963)	0.684 (95% CI: 0.552, 0.791)	0.178
AdaBoost	0.709 (95% CI: 0.587, 0.837)	0.750 (95% CI: 0.660, 0.830)	0.545 (95% CI: 0.379, 0.725)	0.851 (95% CI: 0.767, 0.930)	0.643 (95% CI: 0.458, 0.815)	0.792 (95% CI: 0.692, 0.881)	0.590 (95% CI: 0.428, 0.724)	0.498
Random Forest (RF)	0.815 (95% CI: 0.718, 0.909)	0.820 (95% CI: 0.750, 0.890)	0.697 (95% CI: 0.531, 0.861)	0.881 (95% CI: 0.800, 0.952)	0.742 (95% CI: 0.581, 0.893)	0.855 (95% CI: 0.769, 0.936)	0.719 (95% CI: 0.576, 0.829)	0.4
Logistic Regression (LR)	0.806 (95% CI: 0.697, 0.899)	0.820 (95% CI: 0.750, 0.890)	0.636 (95% CI: 0.481, 0.800)	0.910 (95% CI: 0.838, 0.971)	0.778 (95% CI: 0.609, 0.926)	0.836 (95% CI: 0.750, 0.917)	0.700 (95% CI: 0.571, 0.829)	0.5
XGBoost	0.756 (95% CI: 0.648, 0.855)	0.740 (95% CI: 0.650, 0.830)	0.848 (95% CI: 0.727, 0.969)	0.687 (95% CI: 0.576, 0.794)	0.571 (95% CI: 0.431, 0.711)	0.902 (95% CI: 0.820, 0.979)	0.683 (95% CI: 0.560, 0.783)	0.328
kNN	0.742 (95% CI: 0.642, 0.842)	0.670 (95% CI: 0.570, 0.770)	0.697 (95% CI: 0.513, 0.850)	0.657 (95% CI: 0.533, 0.770)	0.500 (95% CI: 0.341, 0.640)	0.815 (95% CI: 0.700, 0.912)	0.582 (95% CI: 0.436, 0.703)	0.4
MLP	0.731 (95% CI: 0.625, 0.838)	0.700 (95% CI: 0.610, 0.790)	0.758 (95% CI: 0.600, 0.897)	0.672 (95% CI: 0.554, 0.784)	0.532 (95% CI: 0.388, 0.667)	0.849 (95% CI: 0.744, 0.936)	0.625 (95% CI: 0.493, 0.732)	0.026
CatBoost	0.848 (95% CI: 0.766, 0.926)	0.830 (95% CI: 0.750, 0.910)	0.758 (95% CI: 0.586, 0.897)	0.866 (95% CI: 0.784, 0.943)	0.735 (95% CI: 0.577, 0.879)	0.879 (95% CI: 0.797, 0.954)	0.746 (95% CI: 0.620, 0.853)	0.235

**Figure 8:**
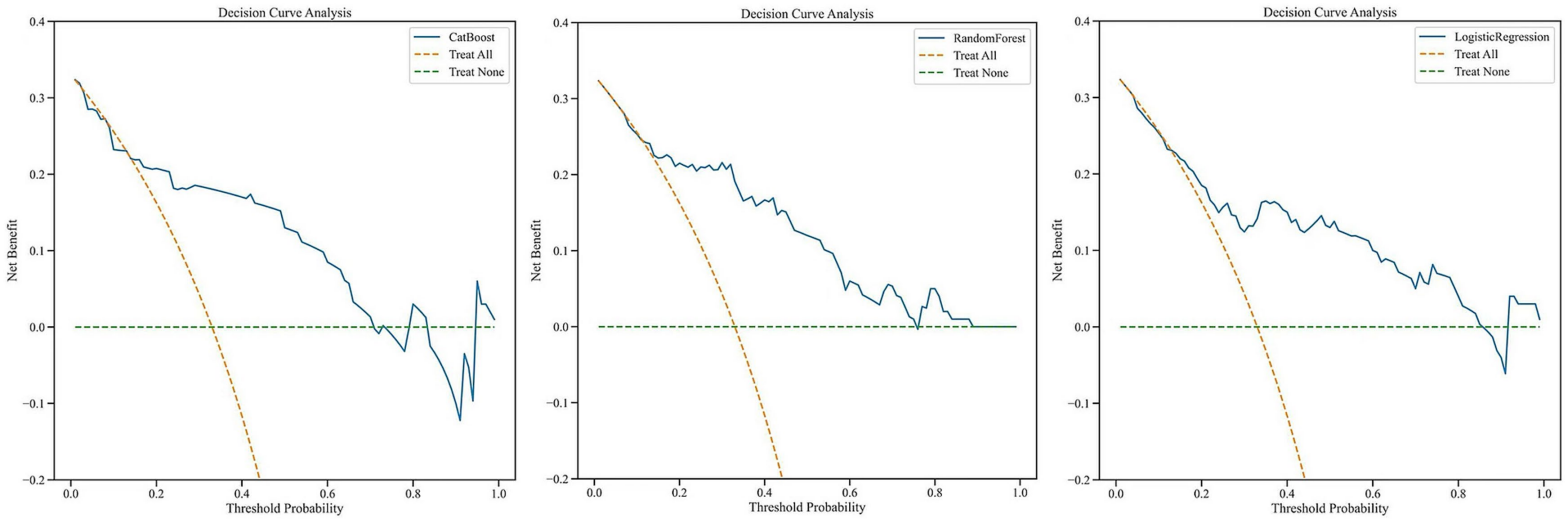
Decision curve analysis of the three top-performing models on the test set.

Decision curve analysis in the test set showed that, across an approximate threshold probability range of 0.10–0.60, the net benefit of the CatBoost model consistently exceeded that of the other top-performing models (Figure 8), indicating greater potential clinical decision-making value. Taken together, these findings support CatBoost as the preferred model, with the best overall balance of discrimination and clinical utility for subsequent application.

### 3.3 Model explanation

**Figure 9:**
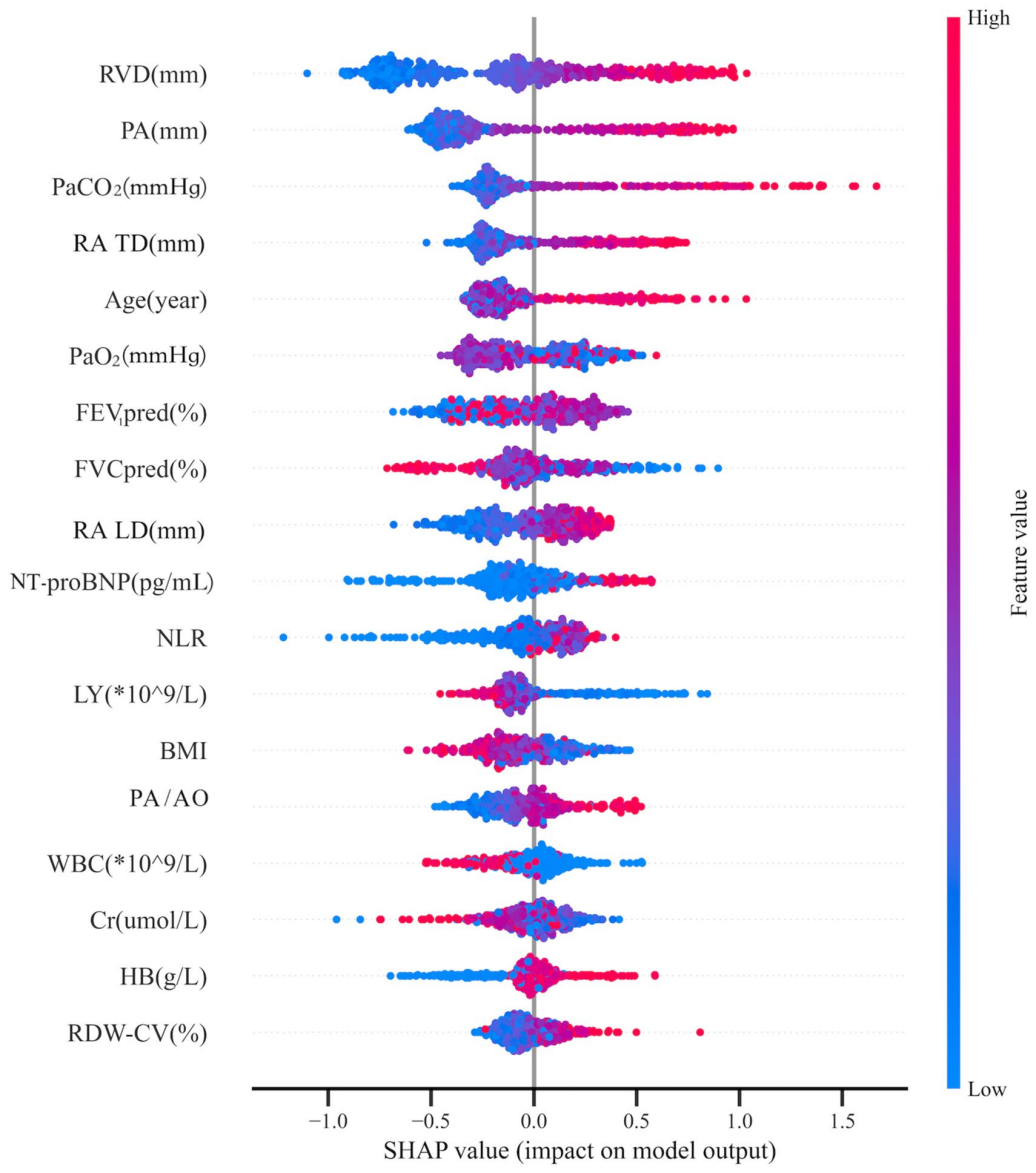
Features ranked by mean absolute SHAP values.

We used SHAP to interrogate the CatBoost model and quantify feature-level contributions at both cohort and individual levels. In the global importance plot (Figure 9), right ventricular diameter (RVD) and pulmonary artery diameter were the dominant structural predictors. Arterial blood gases (PaCO₂, PaO₂) reflected gas-exchange impairment, while right atrial transverse and longitudinal diameters (RA TD, RA LD) captured right-sided cardiac remodeling. NT-proBNP and the neutrophil-to-lymphocyte ratio (NLR) indexed cardiac strain and systemic inflammation. Spirometric indices (FEV₁/FVC, FEV₁% predicted), hematologic markers (white blood cell and lymphocyte counts, hemoglobin, RDW-CV), and additional clinical or imaging variables (creatinine, BMI, PA/AO) contributed additional, though more modest, discriminatory information.

**Figure 10:**
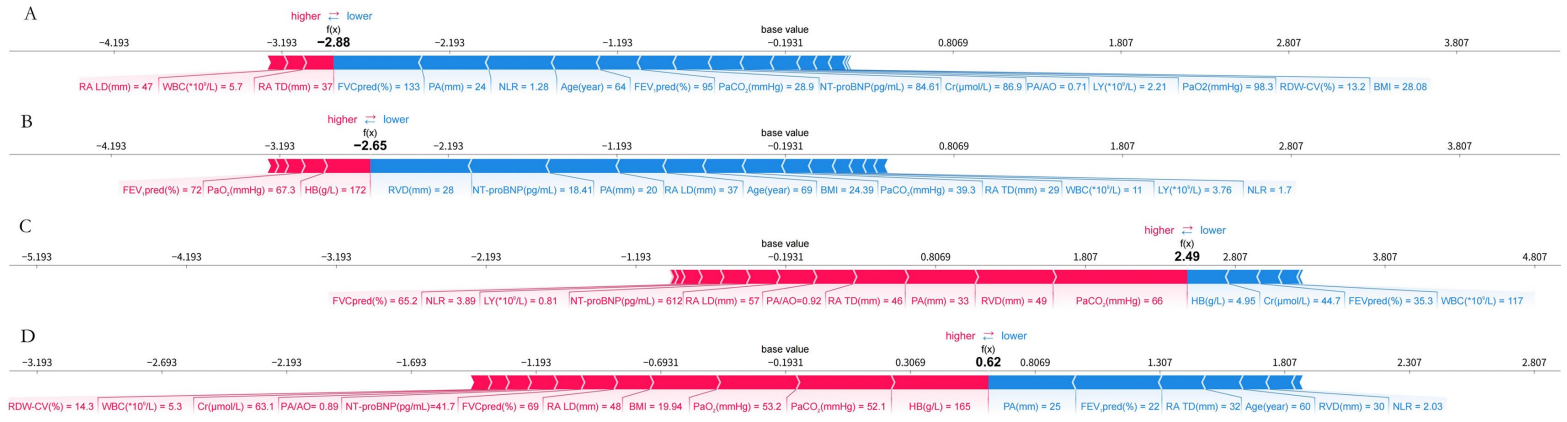
Case-level SHAP explanations for GOLD 1–4 exemplars. **(A)** GOLD 1 exemplar. **(B)** GOLD 2 exemplar. **(C)** GOLD 3 exemplar. **(D)** GOLD 4 exemplar. In each panel, features with bars extending to the right increase, and those extending to the left decrease, the predicted PH risk.

Figure 10 illustrates case-level feature attributions for representative patients across GOLD 1–4 airflow limitation categories. In the GOLD 1 exemplar, enlarged right atrial diameters (RA TD 37 mm, RA LD 47 mm) and a white blood cell count of 5.7 × 10^9^/L have positive SHAP contributions, shifting the prediction toward higher PH risk, whereas a supranormal FVC % predicted (133%) and a relatively small main pulmonary artery diameter (24 mm) have negative SHAP contributions that attenuate the overall risk estimate.

**Figure 11:**
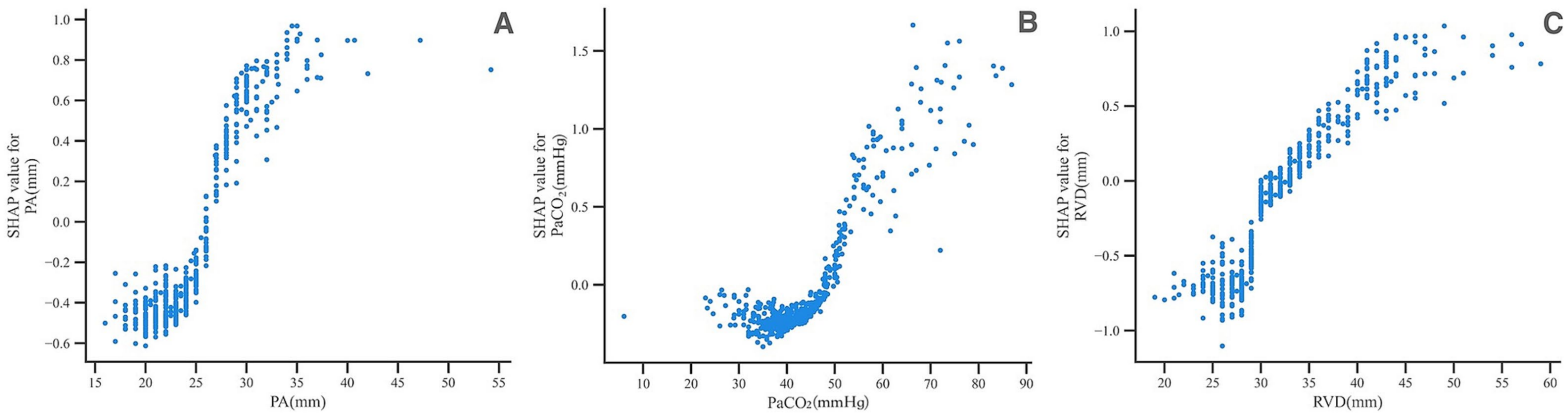
SHAP dependence plots for key predictors. **(A)** Dependence of SHAP values on pulmonary artery diameter. **(B)** Dependence of SHAP values on PaCO_2_. **(C)** Dependence of SHAP values on right ventricular diameter. Each point represents one patient, with the *x*-axis showing the raw feature value and the *y*-axis the corresponding SHAP value; higher SHAP values indicate greater contribution to the predicted PH risk.

Figure 11 (SHAP dependence plots) highlights clinically interpretable regions in which the model’s predicted PH risk increases, such as pulmonary artery diameter > 25 mm, PaCO_2_ > 50 mm Hg, and RVD > 30 mm. These values are not intended as prespecified clinical cut-offs but rather as data-driven inflection points that may inform triage decisions and generate hypotheses for future studies.

**Figure 12:**
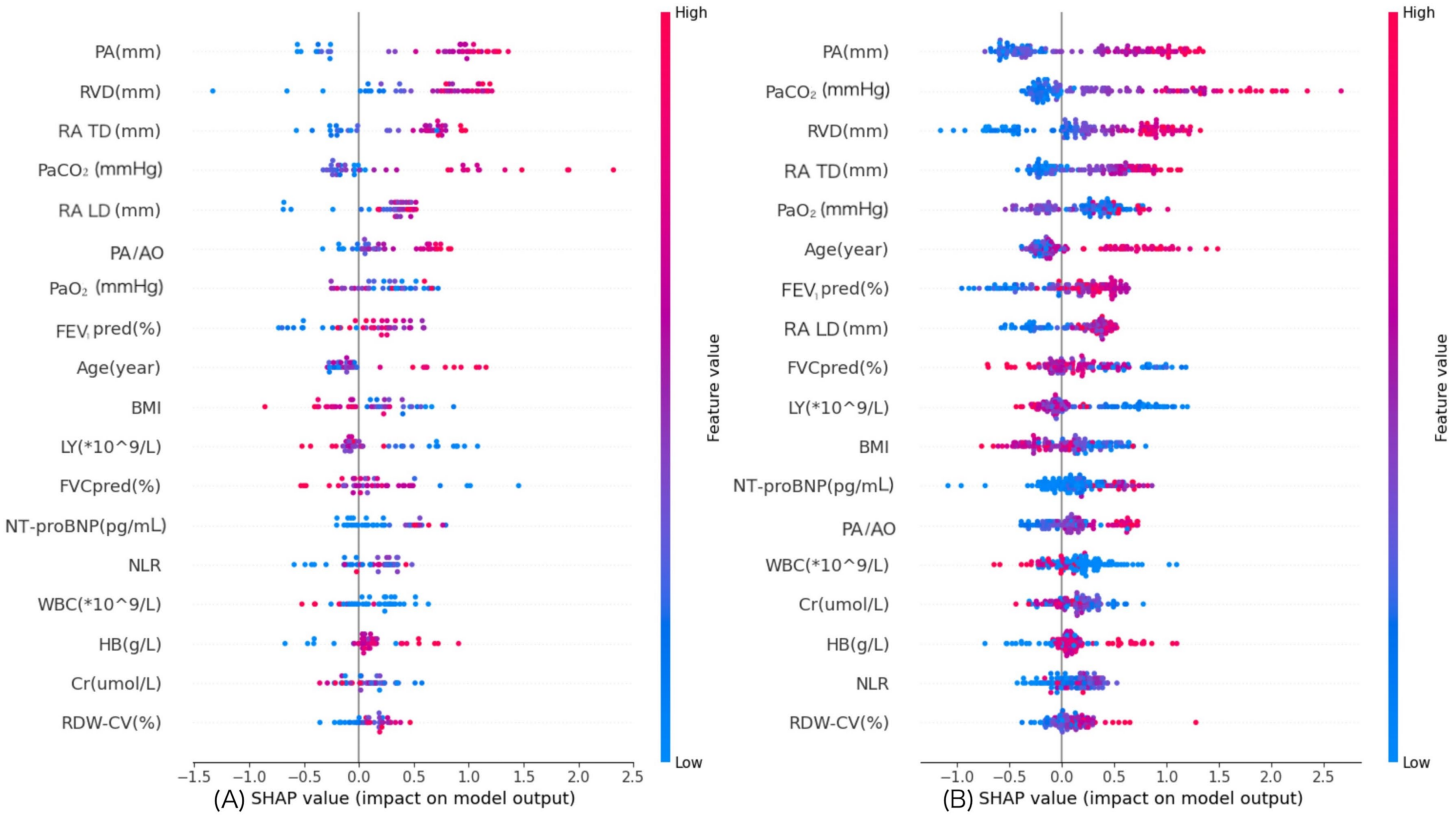
SHAP summary plot. **(A)** SHAP summary plot for the RHC-diagnosed PH subgroup. **(B)** SHAP summary plot for the echo-diagnosed PH subgroup.

Interpretability analyses using subgroup SHAP summaries suggested broadly consistent global feature-importance patterns between the RHC-diagnosed and echo-diagnosed subgroups (Figure 12). Because the RHC-diagnosed subgroup was small, subgroup-specific discrimination metrics were considered exploratory and were not emphasized; we therefore focused on the consistency of model explanations using subgroup SHAP summaries.

## 4 Discussion

### 4.1 Model performance

We developed and internally validated a machine-learning model to estimate PH risk in patients with COPD. Among the eight candidate algorithms, CatBoost provided the strongest overall discrimination and was therefore selected as the final model for downstream evaluation and interpretation.

In clinical risk stratification, CatBoost offers multiple practical advantages: it supports the handling of categorical variables within electronic health records, effectively mitigates overfitting through its symmetric tree structure and ordered boosting mechanism (30), and integrates multi-modal data sources without requiring complex feature engineering (31). Furthermore, its built-in missing value handling strategy enhances model robustness in real-world datasets containing incomplete observations, establishing CatBoost as a pragmatic and reliable choice for clinical decision support applications (32).

### 4.2 Key predictive factors

Based on SHAP feature ranking of the CatBoost model, the five most important predictors were right ventricular diameter, pulmonary artery diameter, arterial partial pressure of carbon dioxide, right atrial transverse diameter, and age. Together, these variables give information about demographic characteristics, heart structure, and breathing function in patients with chronic obstructive pulmonary disease and pulmonary hypertension (COPD-PH).

#### 4.2.1 Right ventricular diameter

Injury to the pulmonary blood vessels, such as hypoxic vasoconstriction, loss of vascular bed, and endothelial dysfunction, raises PVR, PAP, and right ventricular afterload. Over time, the right ventricle shifts from an adaptive to a maladaptive state, with increased right ventricular diameter reflecting this transition (33, 34).

#### 4.2.2 Pulmonary artery diameter

Hypoxemia causes the pulmonary artery to constrict by triggering cytokine release and production of reactive oxygen species (ROS). These changes increase pulmonary vascular resistance and then lead to enlargement of the pulmonary artery diameter (35).

#### 4.2.3 Carbon dioxide partial pressure

Hypercapnia speeds up the progression of PH. It causes pulmonary vasoconstriction and promotes injury and remodeling of the pulmonary vascular endothelium, which increases pulmonary vascular resistance (36).

#### 4.2.4 Right atrial transverse diameter

When pulmonary vascular resistance rises, right ventricular afterload also rises. The right atrium then dilates as a compensatory response. As the disease gets worse, the right atrium becomes larger, right ventricular diastolic function declines, and atrial remodeling increases (37).

#### 4.2.5 Age

In older patients, changes in lung tissue reduce the elasticity of the pulmonary vessels. At the same time, long-term systemic inflammation in this group further harms pulmonary vascular endothelial function and raises pulmonary artery pressure. These effects speed up both the start and the worsening of COPD-PH (38, 39).

### 4.3 Strengths of the study

#### 4.3.1 Data scope

This study used standard diagnostic criteria and one consistent data collection process. We enrolled 523 patients. This sample gives enough power to assess COPD-PH risk inside the cohort and to find clinically important correlations. The candidate predictors cover main areas: demographic characteristics, clinical history, laboratory indicators, pulmonary function, and echocardiography. Together, these variables give a broad description of the COPD-PH phenotype.

#### 4.3.2 Data analysis and interpretability

We compared several machine learning models in a systematic way, and we chose the CatBoost model because it performed best on this mixed clinical data. The explanations from SHAP are in line with known pathophysiological findings in COPD-PH.

#### 4.3.3 Practical application potential

The final model uses variables that are routinely collected in clinical practice and gives explanations at the level of each patient. This makes it suitable for real-world use. It can work as a simple risk calculator or be built into decision support tools in electronic health record systems.

### 4.4 Limitations

This study has several limitations. First, the single-center, retrospective design may introduce selection bias, information bias, and unmeasured confounding. Second, we did not perform external validation; therefore, transportability to other case-mix profiles, imaging protocols, and laboratory platforms remains uncertain and should be tested prospectively. Third, PH was primarily ascertained by echocardiography; although appropriate for screening, it may misclassify cases compared with RHC, the hemodynamic gold standard. The RHC-diagnosed subgroup was small, limiting the precision of subgroup-specific estimates; thus, subgroup findings were considered descriptive/exploratory and warrant confirmation in larger cohorts with broader RHC verification. Finally, some clinically relevant candidate predictors—most notably DLCO—were excluded because of substantial missingness under our prespecified ≥30% threshold, which may have limited model generalizability; future studies with standardized diffusion testing should evaluate the incremental value of DLCO.

### 4.5 Future directions

We plan to improve the model so that it can be built into the electronic medical record (EMR). In this setting, the system will automatically pull routinely collected variables and will ask clinicians to add any missing key predictors before it estimates risk. When non-critical predictors are missing or seem unlikely, the system should mark these values for checking and either ask for confirmation or continue using only the other confirmed predictors. The main users will be pulmonology and cardiology specialists and residents who manage COPD-PH. They will not need special machine learning skills, only a clear understanding of the COPD-PH risk probabilities and the related risk groups given by the model.

Future work should focus on multicenter collaborative datasets and prospective external validation. This will test how well the model works in different patient groups, imaging protocols, and laboratory platforms. Adding more cases confirmed by right heart catheterization would also improve diagnostic accuracy for pulmonary hypertension. In addition, future studies should examine how model-based risk stratification changes daily clinical care, including diagnostic test requests, referral patterns, treatment choices, and patient-centered outcomes. Ideally, the model should be tested in prospective trials that follow real clinical pathways in different healthcare systems.

## 5 Conclusion

Based on routinely collected clinical data, we developed and internally tested a CatBoost model to detect COPD-PH. The model reached an area under the curve (AUC) of 0.848. Decision curve analysis showed that the model gives a net clinical benefit. The main predictive variables were right ventricular diameter, pulmonary artery diameter, arterial partial pressure of carbon dioxide, right atrial transverse diameter, and age. These variables cover structural, hemodynamic, ventilatory, and demographic domains. They match known pathophysiological features of COPD-PH and support bedside risk stratification.

In the future, we plan to test how well the model works in different centers and how it affects clinical outcomes, using multicenter external validation and prospective effectiveness studies. We expect to build the model into electronic health record systems and to create a web-based risk calculator to improve triage for echocardiography and right heart catheterization. This may help make better use of right heart monitoring resources and support more personalized COPD care.

## Patient and public involvement

Patients and members of the public were not involved in the design, conduct, reporting, or dissemination plans of this research.

## Data Availability

The raw data supporting the conclusions of this article will be made available by the authors, without undue reservation.
