# Supplementary material for: Development and internal validation of a machine learning–based prediction model for pulmonary hypertension in COPD
Source: Front Med (Lausanne). 2026 Feb 18;13:1752113. doi: 10.3389/fmed.2026.1752113 (PMC12956692; doi:10.3389/fmed.2026.1752113)
Supplement: Supplementary file 1 [file Data_Sheet_1.docx]

Supplementary Material

# Supplementary Tables

Supplementary Table 1. Missing value information.

| **Feature** | **Missing Value, n (%)** |
| --- | --- |
| **Age(year)** | 2(0.38) |
| **Height(cm)** | 14(2.68) |
| **Body weight(kg)** | 20(3.82) |
| **BMI** | 19(3.63) |
| **Smoking index** | 0(0) |
| **Smoking cessation(year)** | 0(0) |
| **Diabetes Mellitus** | 0(0) |
| **Hyperlipidemia** | 0(0) |
| **Gender** | 0(0) |
| **PaO_2_(mm Hg)** | 54(10.33) |
| **PaCO_2_(mm Hg)** | 54(10.33) |
| **Cr(μmol/L)** | 10(1.91) |
| **UA (μmol/L)** | 11(2.1) |
| **ALB(g/L)** | 12(2.29) |
| **CRP (mg/dL)** | 30(5.74) |
| **NT-proBNP(pg/mL)** | 104(19.89) |
| **ESR (mm/h)** | 76(14.53) |
| **MPV (fL)** | 10(1.91) |
| **RDW-CV (%)** | 9(1.72) |
| **PLT(×10^9^/L)** | 2(0.38) |
| **WBC(×10^9^/L)** | 2(0.38) |
| **HB(g/L)** | 2(0.38) |
| **NE (×10^9^/L)** | 3(0.57) |
| **LY(×10^9^/L)** | 9(1.72) |
| **NLR** | 9(1.72) |
| **FEV_1_pred (%)** | 95(18.16) |
| **FVCpred (%)** | 104(19.89) |
| **FEV_1_/FVC** | 95(18.16) |
| **DLCO SB (mmol/min/kPa)** | 155(29.64) |
| **RA TD (mm)** | 31(5.93) |
| **RA LD (mm)** | 31(5.93) |
| **RVD (mm)** | 31(5.93) |
| **PA (mm)** | 39(7.46) |
| **EF (%)** | 31(5.93) |
| **IVS (mm)** | 31(5.93) |
| **AAo(mm)** | 31(5.93) |
| **PVmax(cm/s)** | 39(7.46) |
| **PA/AO** | 41(7.84) |

Supplementary Table 2. Model-specific hyperparameters for the machine learning algorithms.

| **Machine learning models** | **parameter setting** | **Parameter setting value** |
| --- | --- | --- |
| **GBM** | ccp alpha | 0 |
|  | criterion | 'friedman mse' |
|  | init | None |
|  | learning rate | 0.01 |
|  | loss | 'log loss' |
|  | max depth | 5 |
|  | max features | None |
|  | max leaf nodes | None |
|  | min impurity decrease | 0 |
|  | min samples leaf | 1 |
|  | min samples split | 2 |
|  | min weight fraction leaf | 0 |
|  | n estimators | 100 |
|  | n iter no change | None |
|  | random state | 42 |
|  | subsample | 0.8 |
|  | tol | 0.0001 |
|  | validation fraction | 0.1 |
|  | verbose | 0 |
|  | warm start | FALSE |
| **AdaBoost** | algorithm | 'SAMME.R' |
|  | estimator | None |
|  | learning rate | 0.5 |
|  | n estimators | 200 |
|  | random state | 42 |
| **Random Forest** | bootstrap | TRUE |
|  | ccp alpha | 0 |
|  | class weight | None |
|  | criterion | 'gini' |
|  | max depth | 10 |
|  | max features | 2 |
|  | max leaf nodes | None |
|  | max samples | None |
|  | min impurity decrease | 0 |
|  | min samples leaf | 1 |
|  | min samples split | 4 |
|  | min weight fraction leaf | 0 |
|  | monotonic cst | None |
|  | n estimators | 100 |
|  | n jobs | None |
|  | oob score | FALSE |
|  | random state | 42 |
|  | verbose | 0 |
|  | warm start | FALSE |
| **Logistic Regression** | C | 0.5 |
|  | class weight | None |
|  | dual | FALSE |
|  | fit intercept | TRUE |
|  | intercept scaling | 1 |
|  | l1 ratio | None |
|  | max iter | 100 |
|  | multi class | 'auto' |
|  | n jobs | None |
|  | penalty | 'l2' |
|  | random state | 42 |
|  | solver | 'lbfgs' |
|  | tol | 0.0001 |
|  | verbose | 0 |
|  | warm start | FALSE |
| **XGBoost** | objective | 'binary:logistic' |
|  | base score | None |
|  | booster | None |
|  | callbacks | None |
|  | colsample bylevel | None |
|  | colsample bynode | None |
|  | colsample bytree | 0.8 |
|  | device | None |
|  | early stopping rounds | None |
|  | enable categorical | FALSE |
|  | eval metric | 'logloss' |
|  | feature types | None |
|  | gamma | 0.1 |
|  | grow policy | None |
|  | importance type | None |
|  | interaction constraints | None |
|  | learning rate | 0.01 |
|  | max bin | None |
|  | max cat threshold | None |
|  | max cat to onehot | None |
|  | max delta step | None |
|  | max depth | 6 |
|  | max leaves | None |
|  | min child weight | None |
|  | missing | nan |
|  | monotone constraints | None |
|  | multi strategy | None |
|  | n estimators | 2000 |
|  | n jobs | None |
|  | num parallel tree | None |
|  | random state | None |
|  | reg alpha | 0 |
|  | reg lambda | None |
|  | sampling method | None |
|  | scale pos weight | None |
|  | subsample | 0.8 |
|  | tree method | None |
|  | validate parameters | None |
|  | verbosity | None |
|  | use label encoder | FALSE |
| **kNN** | algorithm | 'auto' |
|  | leaf size | 30 |
|  | metric | 'minkowski' |
|  | metric params | None |
|  | n jobs | None |
|  | n neighbors | 5 |
|  | p | 2 |
|  | weights | 'uniform' |
| **MLP** | activation | 'relu' |
|  | alpha | 0.0001 |
|  | batch size | 'auto' |
|  | beta 1 | 0.9 |
|  | beta 2 | 0.999 |
|  | early stopping | FALSE |
|  | epsilon | 0.00000001 |
|  | hidden layer sizes | 100 |
|  | learning rate | 'constant' |
|  | learning rate init | 0.001 |
|  | max fun | 15000 |
|  | max iter | 200 |
|  | momentum | 0.9 |
|  | n iter no change | 10 |
|  | nesterovs momentum | TRUE |
|  | power t | 0.5 |
|  | random state | 42 |
|  | shuffle | TRUE |
|  | solver | 'adam' |
| **CatBoost** | iterations | 500 |
|  | learning rate | 0.03 |
|  | depth | 6 |
|  | l2 leaf reg | 3 |
|  | verbose | 0 |
|  | random state | 42 |

Supplementary Table 3. Baseline characteristics of the training and test sets.

| **Variable** | **Test** | **p-value** | **Train Set Summary** | **Test Set Summary** |
| --- | --- | --- | --- | --- |
| **Gender** | Fisher's Exact | 0.53 | Female: 127 (30.0%) | Female: 26 (26.0%) |
|  |  |  | Male: 296 (70.0%) | Male: 74 (74.0%) |
| **Age(year)** | Mann-Whitney U | 0.94 | 69.00 (63.00, 75.00) | 68.00 (63.75, 75.25) |
| **Height(cm)** | Mann-Whitney U | 0.85 | 165.00 (160.00, 170.00) | 165.00 (159.75, 171.00) |
| **Body weight(kg)** | Mann-Whitney U | 0.35 | 63.00 (56.00, 71.00) | 65.00 (56.00, 74.25) |
| **BMI** | Mann-Whitney U | 0.28 | 23.23 (20.92, 26.07) | 23.91 (21.18, 27.68) |
| **Smoking index** | Mann-Whitney U | 0.02 | 400.00 (0.00, 800.00) | 600.00 (0.00, 1000.00) |
| **Smoking cessation(year)** | Mann-Whitney U | 0.28 | 0.00 (0.00, 5.00) | 0.00 (0.00, 5.25) |
| **Hyperlipidemia** | Fisher's Exact | 1.00 | No: 383 (90.5%) | No: 81 (81.0%) |
|  |  |  | Yes: 40 (9.5%) | Yes: 19 (19.0%) |
| **Diabetes Mellitus** | Fisher's Exact | 0.44 | No: 335 (79.2%) | No: 86 (86.0%) |
|  |  |  | Yes: 88 (20.8%) | Yes: 14 (14.0%) |
| **PaO_2_(mm Hg)** | Mann-Whitney U | 0.64 | 72.60 (63.50, 84.35) | 74.55 (64.75, 83.35) |
| **PaCO_2_(mm Hg)** | Mann-Whitney U | 0.33 | 42.00 (38.35, 48.00) | 41.25 (38.83, 45.78) |
| **Cr(μmol/L)** | Mann-Whitney U | 0.49 | 69.30 (60.35, 81.65) | 68.00 (57.17, 79.30) |
| **UA (μmol/L)** | Mann-Whitney U | 0.91 | 315.10 (246.30, 396.66) | 318.60 (244.50, 396.33) |
| **ALB(g/L)** | Mann-Whitney U | 0.23 | 37.50 (35.05, 39.80) | 37.65 (35.38, 41.00) |
| **CRP (mg/dL)** | Mann-Whitney U | 0.70 | 3.60 (0.53, 7.00) | 5.00 (0.78, 5.00) |
| **NT-proBNP(pg/mL)** | Mann-Whitney U | 0.03 | 162.90 (67.60, 563.12) | 144.25 (51.97, 331.88) |
| **ESR (mm/h)** | Mann-Whitney U | 0.19 | 15.00 (5.00, 30.00) | 10.00 (5.00, 25.00) |
| **MPV (fL)** | Mann-Whitney U | 0.43 | 10.10 (9.40, 10.75) | 9.95 (9.30, 10.70) |
| **RDW-CV (%)** | Mann-Whitney U | 0.23 | 13.40 (12.85, 14.10) | 13.30 (12.80, 13.93) |
| **PLT(****×10^9^/L)** | Mann-Whitney U | 0.80 | 205.00 (165.00, 257.00) | 208.50 (181.00, 235.25) |
| **WBC(×10^9^/L)** | Mann-Whitney U | 0.79 | 7.50 (5.60, 10.50) | 6.93 (5.82, 9.88) |
| **HB(g/L)** | Mann-Whitney U | 0.12 | 129.00 (105.00, 144.00) | 134.50 (114.00, 143.25) |
| **NE(×10^9^/L)** | Mann-Whitney U | 0.58 | 4.04 (3.00, 5.41) | 3.94 (3.29, 5.49) |
| **LY(×10^9^/L)** | Mann-Whitney U | 0.36 | 1.52 (1.13, 1.98) | 1.58 (1.22, 2.01) |
| **EO(×10^9^/L)** | Mann-Whitney U | 0.10 | 0.13 (0.07, 0.22) | 0.16 (0.06, 0.30) |
| **NLR** | Mann-Whitney U | 0.69 | 2.58 (1.82, 3.93) | 2.47 (1.75, 3.89) |
| **FEV_1_pred (%)** | Mann-Whitney U | 0.48 | 57.50 (40.45, 74.92) | 56.10 (35.22, 72.00) |
| **FVCpred (%)** | t-test | 0.91 | 87.54 ± 20.83 | 87.81 ± 19.74 |
| **FEV_1_/FVC** | Mann-Whitney U | 0.29 | 51.52 (39.72, 60.28) | 50.60 (35.00, 61.10) |
| **DLCO SB (mmol/min/kPa)** | t-test | 0.37 | 67.42 ± 20.85 | 65.05 ± 24.26 |
| **RA TD (mm)** | Mann-Whitney U | 0.02 | 33.00 (30.00, 38.00) | 32.00 (29.00, 36.00) |
| **RA LD (mm)** | Mann-Whitney U | 0.16 | 45.00 (41.00, 50.00) | 44.00 (41.00, 48.00) |
| **RVD (mm)** | Mann-Whitney U | 0.04 | 31.00 (28.00, 36.00) | 30.00 (28.00, 34.00) |
| **PA (mm)** | Mann-Whitney U | 0.06 | 24.00 (22.00, 27.00) | 23.00 (21.75, 25.00) |
| **EF (%)** | Mann-Whitney U | 0.50 | 68.00 (64.75, 71.00) | 68.00 (64.00, 70.00) |
| **IVS (mm)** | Mann-Whitney U | 0.46 | 10.00 (9.10, 10.60) | 10.10 (9.40, 10.72) |
| **AAo (mm)** | Mann-Whitney U | 0.06 | 32.00 (30.00, 35.00) | 31.00 (29.00, 34.00) |
| **PVmax (cm/s)** | Mann-Whitney U | 0.10 | 91.00 (79.00, 103.00) | 85.50 (78.00, 99.25) |
| **PA/AO** | Mann-Whitney U | 0.59 | 0.76 (0.69, 0.84) | 0.74 (0.67, 0.85) |
| **Diagnosis COPD-PH** | Fisher's Exact | 0.37 | No: 280 (66.2%) | No: 67 (67.0%) |
|  |  |  | Yes: 143 (33.8%) | Yes: 33 (33.0%) |

Supplementary Table 4. Baseline characteristics of PH patients by ascertainment method (RHC-confirmed vs Echo-diagnosed).

| **Variable** | **Test** | **p-value** | **Echo-confirmed** | **RHC-confirmed** |
| --- | --- | --- | --- | --- |
| **Gender** | Mann-Whitney U | 0.1023 | 1.00 (0.00, 1.00) | 1.00 (1.00, 1.00) |
| **Age(year)** | t-test | 0.3703 | 70.50 ± 9.69 | 68.93 ± 10.86 |
| **Height(cm)** | t-test | 0.6791 | 163.92 ± 7.79 | 164.47 ± 6.68 |
| **Body weight(kg)** | t-test | 0.0614 | 63.80 ± 13.29 | 59.57 ± 11.12 |
| **BMI** | Mann-Whitney U | 0.0946 | 23.18 (19.94, 27.34) | 22.04 (19.48, 25.39) |
| **Smoking index** | Mann-Whitney U | 0.1413 | 300.00 (0.00, 800.00) | 400.00 (0.00, 950.00) |
| **Smoking cessation(year)** | Mann-Whitney U | 0.1439 | 0.00 (0.00, 8.00) | 1.00 (0.00, 5.00) |
| **Hyperlipidemia** | Mann-Whitney U | 0.5842 | 0.00 (0.00, 0.00) | 0.00 (0.00, 0.00) |
| **Diabetes Mellitus** | Mann-Whitney U | 0.9797 | 0.00 (0.00, 0.00) | 0.00 (0.00, 0.00) |
| **PaO_2_(mm Hg)** | Mann-Whitney U | 0.0126 | 67.50 (57.50, 88.10) | 59.60 (50.95, 69.92) |
| **PaCO_2_(mm Hg)** | Mann-Whitney U | 0.1152 | 48.00 (40.00, 56.70) | 43.00 (37.10, 55.00) |
| **Cr(μmol/L)** | Mann-Whitney U | 0.0662 | 68.00 (59.40, 80.70) | 77.20 (61.10, 95.90) |
| **UA (μmol/L)** | Mann-Whitney U | 0.0069 | 319.90 (236.00, 394.10) | 379.00 (282.50, 519.50) |
| **ALB(g/L)** | Mann-Whitney U | 0.6212 | 37.00 (34.20, 39.20) | 36.90 (34.90, 39.30) |
| **CRP (mg/dL)** | Mann-Whitney U | 0.2494 | 2.37 (0.50, 5.33) | 1.57 (0.53, 4.53) |
| **NT-proBNP(pg/mL)** | Mann-Whitney U | 0.0224 | 297.04 (83.10, 1211.20) | 665.50 (163.00, 3271.00) |
| **ESR (mm/h)** | Mann-Whitney U | 0.5098 | 13.00 (5.00, 25.00) | 9.00 (5.00, 25.00) |
| **MPV (fL)** | Mann-Whitney U | 0.7815 | 10.30 (9.60, 10.90) | 10.40 (9.60, 11.15) |
| **RDW-CV (%)** | Mann-Whitney U | 0.0273 | 13.70 (12.90, 14.40) | 14.10 (13.15, 15.30) |
| **PLT(10^9^/L)** | Mann-Whitney U | 0.4284 | 193.00 (160.00, 235.00) | 184.00 (142.00, 225.00) |
| **WBC(10^9^/L)** | Mann-Whitney U | 0.9314 | 6.80 (5.30, 11.20) | 7.13 (5.81, 8.90) |
| **HB(g/L)** | Mann-Whitney U | 0.5876 | 130.00 (90.00, 145.00) | 129.00 (112.00, 150.50) |
| **NE(×10^9^/L)** | Mann-Whitney U | 0.5434 | 4.09 (3.12, 5.59) | 4.38 (3.58, 5.54) |
| **LY(×10^9^/L)** | Mann-Whitney U | 0.4375 | 1.37 (0.93, 1.87) | 1.27 (0.95, 1.70) |
| **EO(×10^9^/L)** | Mann-Whitney U | 0.9341 | 0.12 (0.06, 0.20) | 0.12 (0.07, 0.22) |
| **NLR** | Mann-Whitney U | 0.2295 | 2.97 (2.11, 4.63) | 3.82 (2.15, 5.17) |
| **FEV1pred (%)** | t-test | 0.0807 | 53.77 ± 21.06 | 60.38 ± 22.64 |
| **FVCpred (%)** | t-test | 0.14 | 81.19 ± 21.35 | 86.80 ± 22.34 |
| **FEV_1_/FVC** | t-test | 0.1511 | 49.94 ± 13.70 | 53.29 ± 11.73 |
| **DLCO SB (mmol/min/kPa)** | t-test | 0.0415 | 61.54 ± 20.08 | 53.95 ± 24.01 |
| **RA TD (mm)** | Mann-Whitney U | 0.026 | 37.00 (31.00, 42.00) | 42.00 (33.50, 46.00) |
| **RA LD (mm)** | Mann-Whitney U | 0.0529 | 48.50 (43.00, 53.00) | 51.00 (46.00, 55.50) |
| **RVD (mm)** | Mann-Whitney U | 0.0006 | 34.00 (30.00, 41.00) | 41.00 (35.00, 45.50) |
| **PA (mm)** | Mann-Whitney U | 0.0005 | 26.00 (23.00, 30.00) | 30.00 (26.00, 33.00) |
| **EF (%)** | Mann-Whitney U | 0.9574 | 68.00 (63.00, 70.00) | 67.00 (64.00, 71.50) |
| **IVS (mm)** | Mann-Whitney U | 0.0219 | 10.00 (9.30, 11.00) | 10.00 (8.00, 10.05) |
| **AAO (mm)** | t-test | 0.237 | 33.04 ± 4.07 | 33.91 ± 4.50 |
| **Pvmax (cm/s)** | t-test | 0.6037 | 92.33 ± 20.09 | 90.37 ± 25.18 |
| **PO/AO** | Mann-Whitney U | 0.0027 | 0.80 (0.72, 0.89) | 0.89 (0.78, 0.96) |

Supplementary Table 5. Fold-wise performance metrics for each model.

| **Model** | **Fold** | **AUC** | **Accuracy** | **Sensitivity** | **Specificity** | **PPV** | **NPV** | **F1 Score** |
| --- | --- | --- | --- | --- | --- | --- | --- | --- |
| **GBM** | 1 | 0.89 | 0.81 | 0.89 | 0.73 | 0.77 | 0.87 | 0.83 |
|  | 2 | 0.91 | 0.84 | 0.88 | 0.80 | 0.82 | 0.87 | 0.84 |
|  | 3 | 0.85 | 0.79 | 0.80 | 0.77 | 0.78 | 0.80 | 0.79 |
|  | 4 | 0.88 | 0.81 | 0.82 | 0.80 | 0.81 | 0.82 | 0.81 |
|  | 5 | 0.89 | 0.82 | 0.80 | 0.84 | 0.83 | 0.81 | 0.82 |
| **AdaBoost** | 1 | 0.89 | 0.82 | 0.77 | 0.88 | 0.86 | 0.79 | 0.81 |
|  | 2 | 0.88 | 0.79 | 0.86 | 0.71 | 0.75 | 0.83 | 0.80 |
|  | 3 | 0.80 | 0.78 | 0.80 | 0.75 | 0.76 | 0.79 | 0.78 |
|  | 4 | 0.86 | 0.79 | 0.82 | 0.77 | 0.78 | 0.81 | 0.80 |
|  | 5 | 0.90 | 0.85 | 0.86 | 0.84 | 0.84 | 0.85 | 0.85 |
| **Random Forest (RF)** | 1 | 0.90 | 0.86 | 0.88 | 0.84 | 0.84 | 0.87 | 0.86 |
|  | 2 | 0.94 | 0.86 | 0.89 | 0.82 | 0.83 | 0.88 | 0.86 |
|  | 3 | 0.87 | 0.80 | 0.82 | 0.79 | 0.79 | 0.81 | 0.81 |
|  | 4 | 0.92 | 0.84 | 0.80 | 0.88 | 0.87 | 0.82 | 0.83 |
|  | 5 | 0.93 | 0.82 | 0.79 | 0.86 | 0.85 | 0.80 | 0.81 |
| **Logistic Regression (LR)** | 1 | 0.85 | 0.79 | 0.75 | 0.84 | 0.82 | 0.77 | 0.79 |
|  | 2 | 0.87 | 0.82 | 0.88 | 0.77 | 0.79 | 0.86 | 0.83 |
|  | 3 | 0.76 | 0.67 | 0.68 | 0.66 | 0.67 | 0.67 | 0.67 |
|  | 4 | 0.80 | 0.79 | 0.77 | 0.82 | 0.81 | 0.78 | 0.79 |
|  | 5 | 0.81 | 0.71 | 0.63 | 0.79 | 0.74 | 0.68 | 0.68 |
| **XGBoost** | 1 | 0.93 | 0.84 | 0.91 | 0.77 | 0.80 | 0.90 | 0.85 |
|  | 2 | 0.95 | 0.88 | 0.91 | 0.86 | 0.86 | 0.91 | 0.89 |
|  | 3 | 0.87 | 0.82 | 0.86 | 0.79 | 0.80 | 0.85 | 0.83 |
|  | 4 | 0.95 | 0.93 | 0.95 | 0.91 | 0.91 | 0.94 | 0.93 |
|  | 5 | 0.95 | 0.87 | 0.88 | 0.86 | 0.86 | 0.87 | 0.87 |
| **kNN** | 1 | 0.78 | 0.71 | 0.80 | 0.63 | 0.68 | 0.76 | 0.74 |
|  | 2 | 0.78 | 0.71 | 0.82 | 0.59 | 0.67 | 0.77 | 0.74 |
|  | 3 | 0.81 | 0.73 | 0.75 | 0.71 | 0.72 | 0.74 | 0.74 |
|  | 4 | 0.82 | 0.78 | 0.79 | 0.77 | 0.77 | 0.78 | 0.78 |
|  | 5 | 0.78 | 0.74 | 0.75 | 0.73 | 0.74 | 0.75 | 0.74 |
| **MLP** | 1 | 0.77 | 0.63 | 0.88 | 0.39 | 0.59 | 0.76 | 0.71 |
|  | 2 | 0.83 | 0.66 | 0.89 | 0.43 | 0.61 | 0.80 | 0.72 |
|  | 3 | 0.73 | 0.63 | 0.95 | 0.32 | 0.58 | 0.86 | 0.72 |
|  | 4 | 0.77 | 0.75 | 0.66 | 0.84 | 0.80 | 0.71 | 0.73 |
|  | 5 | 0.62 | 0.63 | 0.32 | 0.95 | 0.86 | 0.58 | 0.47 |
| **CatBoost** | 1 | 0.93 | 0.83 | 0.89 | 0.77 | 0.79 | 0.88 | 0.84 |
|  | 2 | 0.96 | 0.90 | 0.91 | 0.89 | 0.89 | 0.91 | 0.90 |
|  | 3 | 0.88 | 0.84 | 0.89 | 0.79 | 0.81 | 0.88 | 0.85 |
|  | 4 | 0.94 | 0.89 | 0.91 | 0.88 | 0.88 | 0.91 | 0.89 |
|  | 5 | 0.95 | 0.88 | 0.86 | 0.91 | 0.91 | 0.86 | 0.88 |
